# Modeling human natural killer cell development and drug response in a microfluidic bone marrow model

**DOI:** 10.3389/fimmu.2025.1499397

**Published:** 2025-02-20

**Authors:** Leopold Koenig, Inbal Ben-Eliezer, Thi Phuong Tao, Annika Winter, Moran Grossman

**Affiliations:** ^1^ Contract Development, TissUse GmbH, Berlin, Germany; ^2^ Non-clinical Development Department, Teva Pharmaceutical Industries Ltd., Netanya, Israel

**Keywords:** organ-on-a-chip, NK cell, bone marrow, drug response, immunotoxicity

## Abstract

**Introduction:**

The human bone marrow is a complex organ that is critical for self-renewal and differentiation of hematopoietic progenitor cells into various lineages of blood cells. Perturbations of the hematopoietic system have been reported to cause numerous diseases. Yet, understanding the fundamental biology of the human bone marrow in health and disease and during the preclinical stages of drug development is challenging due to the complexity of studying or manipulating the human bone marrow. Human cell-based microfluidic bone marrow models are promising research tools to explore multi-lineage differentiation of human stem and progenitor cells over long periods of time.

**Methods:**

Human hematopoietic stem and progenitor cells were cultured with mesenchymal stromal cells on a zirconium oxide ceramic scaffold in a microfluidic device recapitulating the human bone marrow. NK cell differentiation was induced by the application of a lymphoid cultivation medium containing IL-15. The kinetics of differentiation into mature NK cells was traced by flow cytometry over a period of up to seven weeks, and functionality was measured by stimulation with phorbol myristate acetate (PMA) and ionomycin. The effect of an anti-IL-15 monoclonal antibody (TEV-53408) on different NK cell subtypes was tested at different time points.

**Results:**

Our data shows that within 28 days of culture, differentiation into all developmental stages of NK cells was accomplished in this system. Alongside with the NK cells, myeloid cells developed in the system including granulocytes, monocytes and dendritic cells. The differentiated NK cells could be activated after stimulation with PMA and ionomycin indicating the functionality of the cells. Treatment with an anti-IL-15 antibody induced a reduction in proliferation of late-stage NK cells as shown by EdU staining. This led to significantly dose dependent reduction in the number of circulating stage 4 - 6 NK cells in the system after one week of treatment. This effect was partially reversible after a two-week treatment-free period.

**Discussion:**

In summary, the presented model enables investigation of human NK cell development in the bone marrow and provides a basis to study related diseases and drug response effects in a microenvironment that is designed mimic human physiology.

## Introduction

Natural killer (NK) cells are innate lymphoid cells (ILCs) that act as the first line of defense of the innate immune system against a wide range of pathogens and developing tumor cells. They develop from common lymphoid progenitor (CLP) cells in the bone marrow upon signaling induced by SCF, FLT-3L, IL-3, IL-7, and IL-15 (1). NK cells are not only located in the bone marrow and the blood circulation but also reside in secondary organs such as lymphoid tissues, lung, and liver where they express tissue-specific phenotypes (2) with marked heterogeneity, even inside a specific tissue (3). Human NK cell development is often described as a linear model composed of six steps, starting from CD34+ hematopoietic stem and progenitor cells (HSPCs), the earliest precursor (stage 1). Then, immature NK cells (stages 2–3) further differentiate into stage 4, at which point NK cells become CD56^bright^ and start expressing NK cell markers and cytokines and further commit to the conventional NK cell lineage, culminating in terminally mature CD56^dim^ CD16^+^ (stage 5) or CD56^dim^ CD16^+^ CD57^+^ (stage 6) cells (4). CD56^dim^ NK cells make up the majority of circulating NK cells and are characterized by expression of CD16, allowing them to eliminate target cells by antibody-dependent cell-mediated cytotoxicity (5). In contrast, CD56^bright^ NK cells execute their effector function by the production of a diverse set of cytokines, including IFN-γ, TNF-β, GM-CSF, IL-10, and IL-13 (6).

The majority of NK cells in humans differentiate and mature inside the bone marrow in specialized niches (7), although it has been shown that they can differentiate and mature in secondary lymphoid organs as well (8). In the bone marrow, hematopoietic stem cells give rise to lymphoid-primed multipotential progenitors that further differentiate into CLPs, which are the starting point of the lymphoid lineage, including innate lymphoid cells, T cells, and B cells. From here, CLPs progress through multiple stages from NK cell progenitors toward the mature NK cell stages characterized by upregulation of CD56 (9). The process of NK cell development, maturation, activation, and survival is controlled by inflammatory cytokines such as interleukin (IL)–2, IL-12, IL-15, IL-18, IL-21, and type I interferons (10) in the bone marrow and circulation, and recapitulating this process preclinically can be used to investigate the efficacy of new drugs and toxic effects that might occur during NK cell development.

Animal models have been used extensively in the past for modeling the human immune response (11). Even if many fundamental principles of NK cell differentiation and function are preserved between humans and mice, receptor expression patterns differ significantly between the species, and species-specific receptor expression was shown to be linked to NK cell functions (12).

As an alternative to animal models, a wide range of *in vitro* human NK cell assays and models have emerged that allow investigation of NK cell activation (13, 14), cytotoxicity (15, 16), and tumor-killing activity (17, 18) of primary human peripheral NK cells. All these models focus on the functionality of mature NK cells and are used as a two-dimensional (2D) cell-based assay; however, they are not suited to investigate NK cell development and functionality in a physiologically relevant 3D human microenvironment. For example, 2D cell-based assays specifically aiming to characterize NK cell development use cell lines that support NK cell development, such as OP9 or OP9-DL1 lines, derived from newborn mouse bone marrow and EL08.1D2 murine fetal liver stromal cell lines (19). In addition, co-cultures with bone marrow stroma, human splenic fibroblasts, mesenchymal stromal cells (MSCs), and other stromal cell lines such as M2–10B4 and AFT024 have been shown to support NK cell differentiation from CD34^+^ progenitors. Stromal-based cell co-culture methods to generate mature NK cells have shown that these matrices can partially support *in vitro* NK cell development but with impaired expansion of NK cell developmental intermediates (20). Various ILC lineages, including ILC1s, ILC2s, and ILC3, can also be efficiently generated from CD34+ HPCs derived from bone marrow under similar culture conditions (21). While these models provide a controlled environment to study NK cell development, allow for high-throughput screening, and are cost-effective, they often lack the complexity of the human microenvironment and do not accurately mimic the 3D interactions and spatial organization found *in vivo*, potentially affecting cell behavior and differentiation. In addition, while stromal-based co-culture methods can support NK cell development, they often result in impaired expansion of NK cell developmental intermediates, limiting their effectiveness in fully recapitulating NK cell maturation in an *in vivo* environment.

3D systems such as organoids and microphysiological systems may be better suited to mimic the physiological microenvironment. Bone marrow organoids, which are often based on human-induced pluripotent stem cells (hiPSCs), have demonstrated the ability to mediate ILC maturations, even in the absence of cytokine supplementation when matured from CD34+ hematopoietic stem cells (22). Similarly, 3D human bone marrow organoids based on either porous scaffolds or hydrogels have been shown to be particularly effective as cell support matrices due to their similarity to the extracellular matrix (23). Furthermore, human NK cell development and maturation have recently been demonstrated in a preprint article using organoids derived from patient biopsies (24).

Microphysiological systems, on the other hand, offer controllable parameters such as concentration gradients, dynamic stress, and shear forces that can be adjusted by the operator. This flexibility allows for the study of different microenvironmental conditions. These systems have been used as preclinical models to investigate new drugs and toxic effects that may occur in the bone marrow (25). A recent publication described a humanized and immunocompetent organ-on-a-chip model combining a neuroblastoma cell line with primary human NK cells isolated from peripheral blood mononuclear cells. This setup enabled investigation of NK cell infiltration and activation in a tumor microenvironment during a 4-hr coculture period (26). Recently, several human *in vitro* bone marrow models have been developed, which are capable of long-term culture of HSCs (27, 28), differentiation of monocytes and neutrophils (29) or differentiation of monocytes, neutrophils, erythroid cells, and megakaryocytes (30, 31). However, none of these assays describe on-chip differentiation of NK cells.

Here, we describe the application of the HUMIMIC Chip2 bone marrow model for the on-chip differentiation of human NK cells over seven weeks for testing of biologics-induced effects on NK cell lineage differentiation. Overall, this model enables investigation of human NK cell development in the bone marrow and may provide a framework for studying drug responses in a physiologically relevant microenvironment.

## Results

### Culture system setup

**Figure 1:**
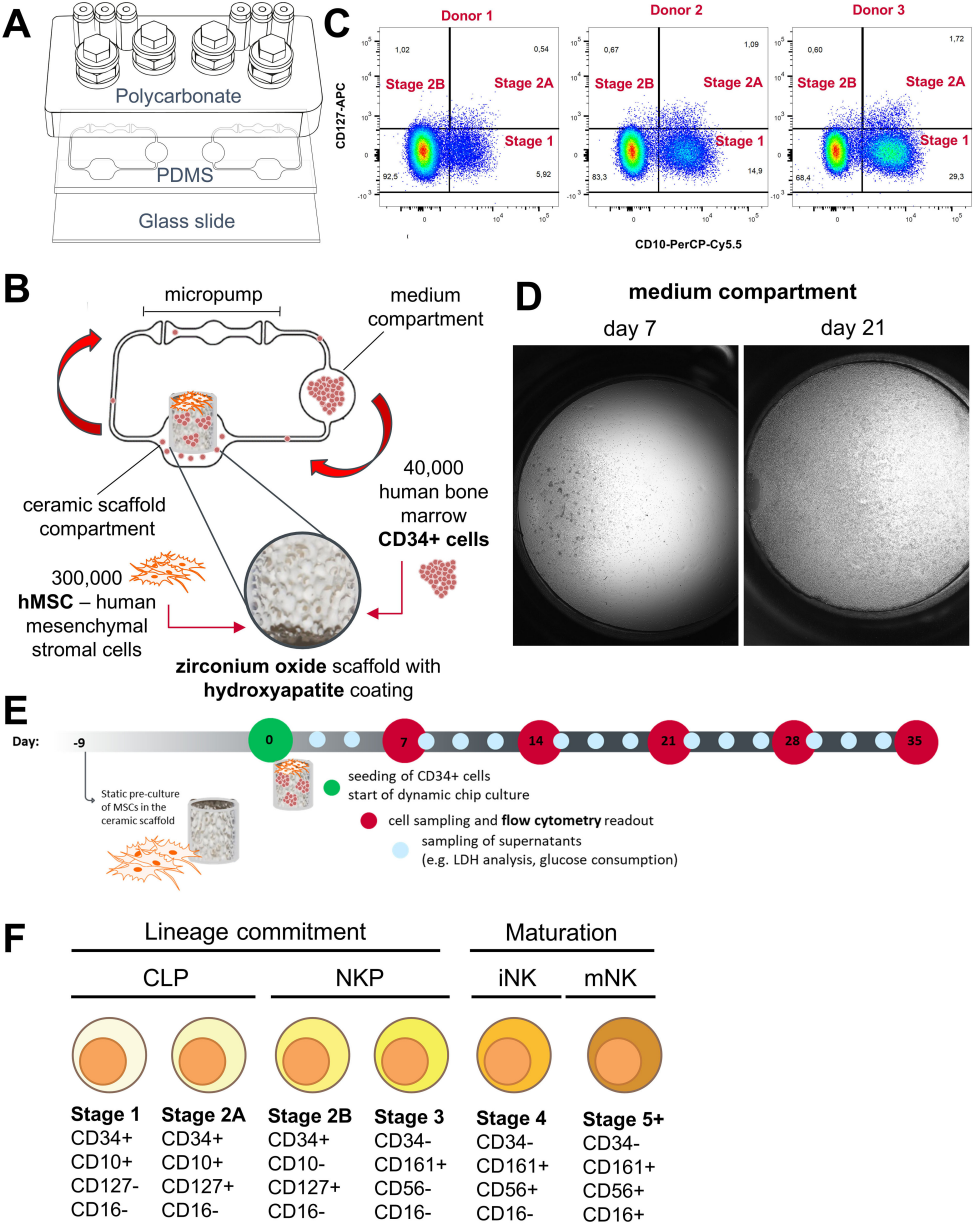
Setup of the NK cell bone marrow model in the HUMIMIC Chip2. **(A)** Exploded view of the HUMIMIC Chip2 showing the three structural layers: the polycarbonate adapter plate housing the culture compartments, the PDMS layer housing the microfluidic circulation, and the bottom glass layer closing the circulation and allowing microscopic observation. **(B)** 2D view of the HUMIMIC Chip2 microfluidics: the outer compartment houses the bone marrow model; the inner compartment is used as a medium compartment and as a sampling point for hematopoietic cells. **(C)** Progenitor populations until Stage 2B of NK cell development in CD34+ starting cell pool from three donors on day 0. **(D)** Exemplary microscopic images of a medium compartment on day 7 with 35% confluency and on day 21 with 100% confluency, scale is 1mm. **(E)** Time schedule of the NK differentiation assay in the Chip2 system with weekly sampling points for FC analysis and media exchanges every 2–3 days. **(F)** Used surface marker to distinguish NK cell maturation stages adapted from Abel et al. (9), Cichoki et al. (33) and Bi et al. (34). NK development goes through a stepwise differentiation starting from hematopoietic stem cells to CLP, NK cell precursors (NKP), and immature NK cells (iNK) toward mature NK cells (mNK).

The NK cell differentiation assay was set up in the HUMIMIC Chip2. The HUMIMIC Chip2 (**Figure 1A**) consists of two independent microfluidic circuits, each consisting of two culture compartments interconnected by a microfluidic channel. Circulation of the cell culture medium is enabled through on-chip micropumps. Cell culture medium is exchanged manually every 2–3 days through the access ports of the culture compartments. The outer culture compartment houses an *in vitro* model of the human bone marrow consisting of a zirconium oxide ceramic scaffold with a hydroxyapatite coating to mimic the architecture of the bone marrow niche. The inner culture compartment is termed the medium compartment and functions as a reservoir for cell culture medium. The bone marrow model was seeded with 300,000 MSCs serving as supporting cells and 40,000 CD34+ HSPCs at the time of seeding (**Figure 1B**). CD34+ presorted cells were used, as CD34 is generally considered a marker for stem and progenitor cells in the hematopoietic lineage that is not present on terminally differentiated cells (32). Stromal cells were seeded onto the ceramics 9 days prior to the initiation of the assay to enable distribution over the scaffold surface, production of extracellular matrix, and induction of an osteogenic phenotype. HSPCs from three different donors were seeded onto the stromal cell–covered scaffold in separate chips and in duplicates and transferred into the microfluidic system on the day the assay was initiated by adding a hematopoietic cell growth medium containing TPO, FLT-3L, and SCF for HSC maintenance and IL-7 and IL-15 for NK cell differentiation. Initial cell pools of CD34+ cells on day 0 after thawing contained considerable amounts stage 1 NK cells (5.92%, 14.9%, and 29.3%) and low amounts of stage 2A and stage 2B NK cell progenitors (**Figure 1C**).

Over time, HSPCs and differentiated cells were transported by the microfluidic circulation into the medium compartment where they accumulated. The number of circulating cells sedimenting in the medium compartment increased over culture time and consistently reached close to 100% confluency (**Figure 1D**). Those cells were sampled weekly from the system and used for counting and flow cytometry (FC) analysis on days 7, 14, 21, 28, and 35 of the assays (**Figure 1E**).

We set up an FC panel containing the early progenitor marker CD34, the lymphoid progenitor marker CD10, the interleukin-7 receptor-α (CD127), the killer cell lectin-like receptor subfamily B, member 1 (CD161), the prototypic NK cell marker (CD56), the type III Fcγ receptor (CD16), the interleukin-2 receptor subunit beta (CD122), the myeloid marker CD33, and the leukocyte common antigen CD45. We analyzed these markers on primary bone marrow–derived mononuclear cells as well as circulating cells from the HUMIMIC Chip2 (**Supplementary Figure 3**). Expression patterns of NK lineage cells in the primary bone marrow–derived mononuclear cells matched for the most part with expression patterns described in literature (9). Contrary to Abel et al. (9), expression of interleukin-7 receptor-α (CD127) was detected until stage 4 of development instead of being downregulated after stage 2B. Minor expression of interleukin-2 receptor subunit beta (CD122) was seen only in the later detected stage 5 but not in the stages before (**Supplementary Figure 3A**). NK cell development on chip showed some differences from these patterns. CD127 expression decreased after stage 2B as described in the literature (9). Events identified as early NK cell progenitors, stages 1 and 2, also expressed the myeloid marker CD33 in the HUMIMIC Chip2 (**Supplementary Figure 3B**). In the second iteration of the FC panel, antibodies detecting CD45 and CD122 were removed from the panel, since they did not help to discriminate different stages. For the final chip assay, events were gated according to the marker combination shown in **Figure 1F** and the gating tree shown in **Supplementary Figure 1**.

**Figure 2:**
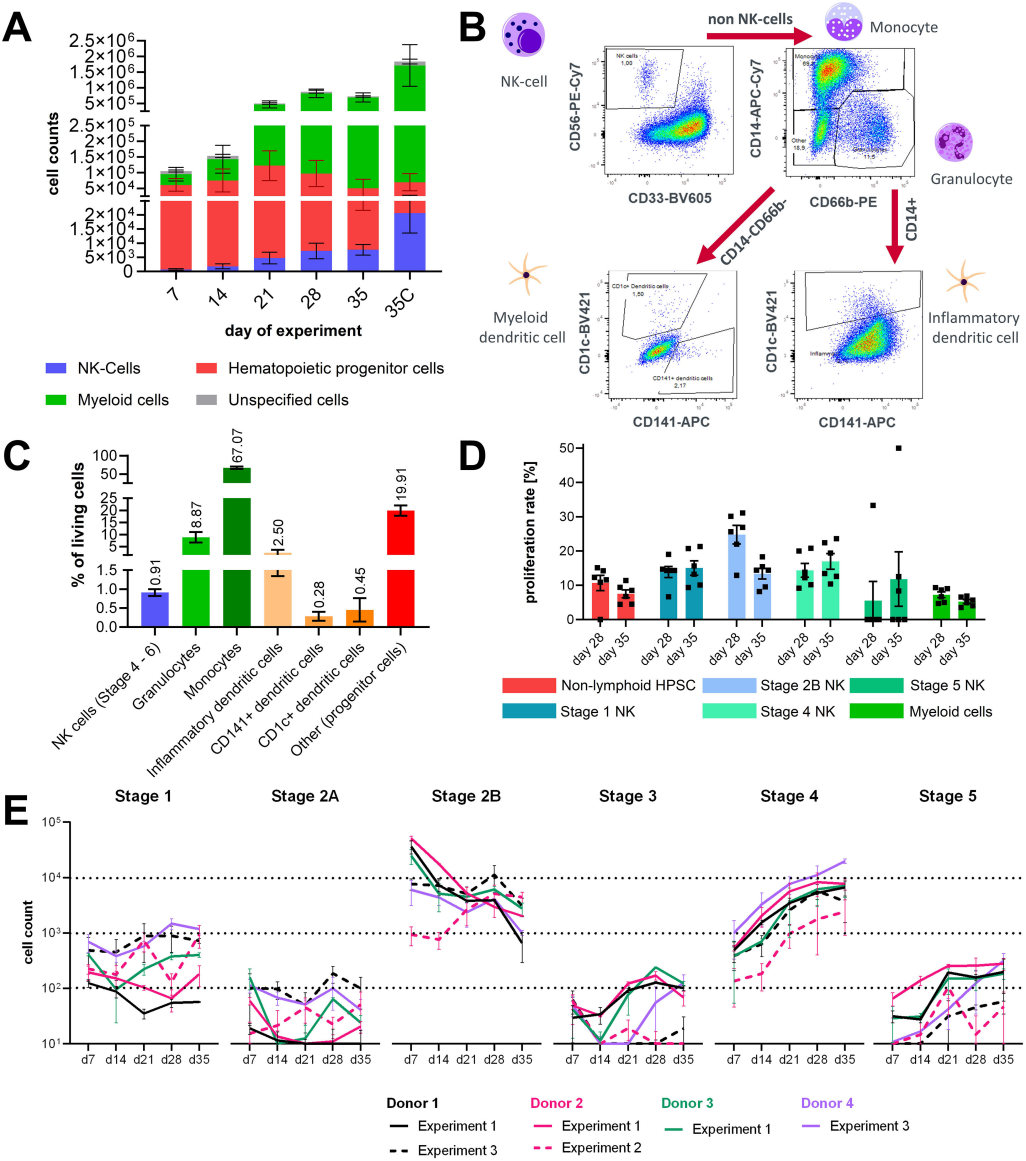
Characterization of lineage differentiation in the bone marrow model. **(A)** Sampled cell counts of circuits over time from the circulation and harvested cell counts form the ceramic scaffold at the end of the experiment. Mean values ± SD of individual cell populations are shown as stacked bar graphs from three CD34+ donors with two chips (N = 3, n = 2). **(B)** Gating strategy for analysis of dendritic cells in the hematopoietic cell pool sampled on day 35 of chip culture. **(C)** Percentage of NK cells, granulocytes, monocyte, inflammatory DCs, myeloid/conventional DC1 and myeloid/conventional DC2 sampled at the end of the assay on day 35. Mean values ± SD of individual cell populations from three CD34+ donors (N = 3, n = 1) are shown. **(D)** Proliferation rate of stage 1, 2, 3, 4, and 5 NK cells in circulation on day 28 and day 35 of the assay. Mean values ± s.e.m of individual cell populations from one CD34+ donors with six chips (N = 1, n = 6) are shown. **(E)** Sampled cell counts of the lymphoid/NK cell lineage from the circulation over time. Mean values ± SD of individual cell populations from three experiments with in total four CD34+ donors with two to three chips (N = 4, n = 2–3) are shown.

Harvested cell counts from the medium compartment increased weekly from approximately 1 × 10^5^ cells on day 7 up to 9 × 10^5^ cells on day 28 and decreased a bit to approximately 7 × 10^5^ cells on day 35 in the first experiments with three different CD34+ donors. Additionally, 18 × 10^5^ cells were harvested from the ceramic scaffold on day 35 of the assay (**Figure 2A**). All three tested donors had a similar cell output. During the first 3 weeks of the experiment, donor 3 had a lower output but reached similar cell numbers as donors 1 and 2 from day 28 on (**Supplementary Figure 1**). Major populations in the analyzed cells throughout the experiment were non-lymphoid HSPCs and myeloid cells and a smaller fraction of NK progenitor cells (**Figure 2A**).

To understand which other cell types co-differentiated alongside the NK cell population, cells from the ceramic scaffold were stained with another FC panel on day 31 of the experiment. Events were gated according to the gating tree shown in **Figure 2B**. Dead events, debris, and cell doublets were removed from the analysis. Then CD56+CD33- negative events were gated as NK cells Stage 4+. Remaining cells were separated based on the expression of CD14 and CD66b into CD14+ cells of the monocyte lineage and CD66b+CD14- granulocytes. Remaining CD14-CD66b- cells were gated for CD1c and CD141+ to select dendritic cells. Dendritic cells were detected in the CD14+ monocyte-lineage population based on CD1c expression (**Figure 2B**). On average, the cultures contained around 0.91% NK cells and 3.23% dendritic cells, from which the majority were dendritic cells. The most prominent cell populations were CD14+ cells labeled as monocytes and CD66b+ granulocytes (**Figure 2C**).

Additional experiments were performed with the same assay setup; in the second experiment CD34+, cell donor 2 was used again, and in the third experiment, CD34+ donor 1 and an additional donor 4 were used. Cell counts of NK cell progenitors and matured NK cells in circulation were analyzed in all experiments at days 7, 14, 21, 28, and 35 of the assays in the untreated control groups. In experiment 2, using CD34+ donor 2, cells were sampled additionally on day 31 of the assay. To allow easy comparison with the other two experiments, sampled cell counts of days 31 and 35 were summarized for **Figure 2E**. Cell counts of NK cell stages 1 and 2A progenitors and stages 3 and 5 in circulation were below 10^3^, which is lower compared to other stages during the whole assay timespan and differed between donors and experiments. Stage 2B progenitors were the largest population among the NK lineage-associated cell types until day 21 but decreased over the course of the experiments. Numbers of stage 4 NK cells in chip circulation continuously increased over the assay timespan and became the biggest population among the NK lineage-associated cell types after day 21. Since increasing numbers of stage 4 cells were seen over the culture time, it can be assumed that cells cycle quickly through stage 3 and that the percentage of cells that are in stage 3 at a given timepoint is extremely small compared to the other stages (**Figure 2E**). During experiment 2, proliferation rates of circulating cells were analyzed at days 28 and 35 of the assay. Proliferation rates of NK cell stages 1, 2B, and 4 ranged from 10% to 30% and were on average higher than those of non-lymphoid HSPCs, stage 5 NK cells, and myeloid cells. Sampled counts of stages 2A and 3 NK cell progenitors were too low to reliably determine their proliferation rate. For stage 2B cells, a marked decrease of proliferation was observed between days 28 and 35, suggesting that the proliferation rate of early stage NK progenitors declines in the presence of increasing numbers of later stage NK cells (**Figure 2D**).

Overall, within 28 days of culture, differentiation into the first five developmental stages of NK cells was accomplished in this system.

### IL-15 affects maturation of NK cells

**Figure 3:**
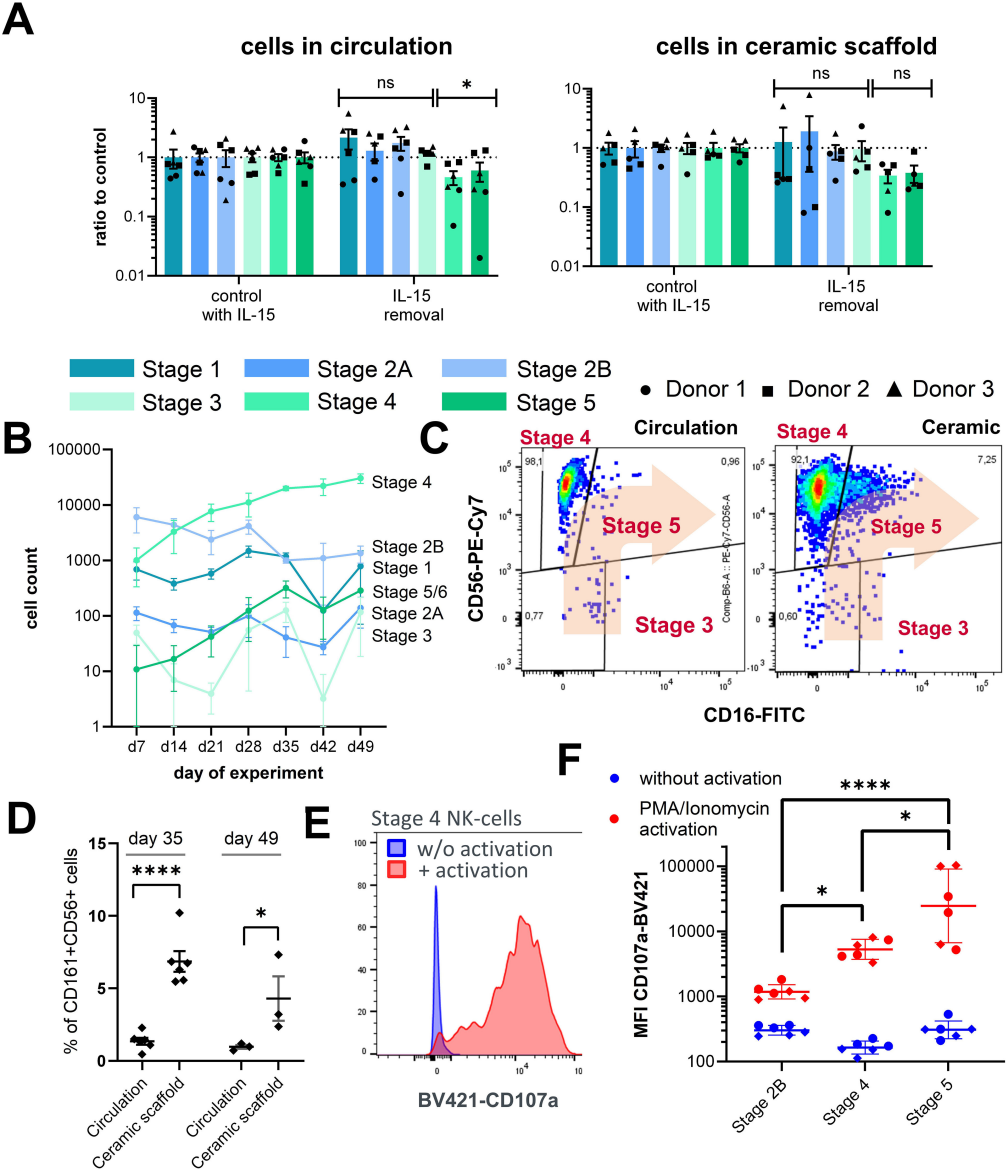
Characterization of NK cell populations upon perturbation of culture parameters. **(A)** Effects of IL-15 removal from the cell culture medium between day 28 and day 35 on stage specific NK cell counts in circulation and in the ceramic scaffold relative to control circuits with continuous IL-15 supplementation. Mean values ± s.e.m of three donors (N = 3) with two chips (n = 2) are shown. Comparison of early (stages 1–3) and late (stages 4–6) NK cell counts by Welch’s t-test with Holm-Sidak correction, n(circulation) = 6, n(ceramic) = 5, *p < 0.05 and ns (p > 0.05). **(B)** Sampled cell counts of the lymphoid/NK cell lineage from the circulation over a time frame of 49 days. Mean values ± SD of individual cell populations are shown as stacked bar graphs from one CD34+ donor with three chips (N = 1, n = 3). **(C)** Exemplary FC plots of CD56 and CD16 expression in CD161+ cells in the circulation and in the ceramic scaffold, used for gating of stages 3, 4, and 5 NK cell populations on day 49 of the assay. **(D)** Fraction of CD16+ cells (stage 5 NK cells) of all CD161+ CD56+ NK cells at days 35 and 49 sampled from the circulation or harvested form the ceramic scaffold. Individual chips and mean ± s.e.m are shown (N = 2, n = 3). Repeated measures mixed effects model REML and Sidak multiple comparisons test, n(day 35) = 6, n(day 49) = 3, ****p < 0.0001 and *p < 0.05. **(E)** Exemplary FC plot of CD107a expression on stage 4 NK cells with and without PMA/ionomycin stimulation **(F)** Mean fluorescence intensity of CD107a-BV421 on stages 2B, 4, and 5 NK cells on day 35 with and without PMA/ionomycin stimulation. Chip-specific intensity values and geometric mean ± SD are shown from one experiment with two CD34+ donor with three chips (N = 2, n = 3). Log transformed MFI values were compared by one-way analysis of variance (ANOVA) and Tukey multiple comparisons test, n = 6, ****p < 0.0001 and *p < 0.05.

To understand the role of IL-15 in the differentiation of NK cell progenitors in this system, IL-15 was withdrawn from the chips after day 28. A decrease in the NK cell counts of stages 4 and 5 NK was detected both in circulation as well as in the ceramic scaffold compared to circuits with continuous IL-15 supplementation. The counts of stages 1–3 were not affected by the removal of IL-15. This is consistent with literature reports of the expression profiles of CD122 (35). Some donor differences were observed for the reduction of stages 4 and 5 NK cells upon IL-15 depletion, especially for the counts of circulating cells. Numbers of stage 4 NK cells derived from donor 2 only showed minor changes upon IL-15 removal, while cells derived from donor 1 were strongly decreased in circuits without constant IL-15 supplementation (**Figure 3A**). This result supports the proposed role of IL-15 in the maturation of NK cells and that the chip system recapitulates this process.

### Culture conditions allow cultivation over a period of seven weeks

Until day 28, no signs of exhaustion of the differentiation capacity based on the increasing numbers of cells in circulation were overserved; therefore, a follow-up experiment with a new CD34+ cell donor was conducted, increasing the total cultivation time to 49 days. After day 28, harvested cell numbers of NK progenitors stages 1 and 2B from circulation declined, while sampled numbers of stages 4–5 NK cells continuously increased until day 49 of the assay (**Figure 3B**). At this timepoint, a noticeable population of stage 5 NK cells was detected in the cell fraction residing inside the ceramic scaffold; however, only a few stage 5 NK cells were visible in circulation. (**Figure 3C**). Consequently, the proportion of CD16+ stage 5 NK cells of all CD161+CD56+ NK cells was significantly higher in the ceramic scaffold cell fraction compared to the circulating cells on both day 35 and day 49 of the assay (**Figure 3D**). Overall, this result indicates that cultivation conditions allow maintenance of NK cells and kinetic tracing of their growth over a duration of 7 weeks and that the system is suitable for long-term culture of NK cells.

### Differentiated NK cells are functional

To test whether NK cells from circulation can be activated, samples from the circulating cell pools were stimulated by phorbol-12-myristat-13-acetat (PMA) and ionomycin to induce lysosomal-associated membrane protein-1 (CD107a) expression on the cell surface, which is a marker for NK cell activation and degranulation (**Figure 3E**).

Upregulation of CD107a cell surface expression upon PMA/ionomycin stimulation was detected on all populations with increasing expression strength from stage 2B to stage 5 (**Figure 3F**). We also analyzed CD107a expression of stages 1 and 2A NK cells, which should not display pronounced degranulation activity, as this is linked to cytotoxic functions. Unexpectedly, these cells also showed potent activation behavior similar to stage 5 NK cells (**Supplementary Figure 4A**). However, when closer examining the expression pattern of CD107a on these cells we found that CD107a upregulation was mostly associated with a CD10^low^ subpopulation among the stages 1 and 2A events, while cells expressing high levels of CD10 only showed low levels of CD107a expression, indicating that the FC-based separation of populations could be improved for future experiments.

Overall, the results obtained confirmed that the established microfluidic bone marrow model supports differentiation into most subpopulations of NK cells and that IL-15 promotes maturation. We also show that maturing NK cells can be activated.

### Drug response

To demonstrate that the system is capable of detecting a drug response, the bone marrow system was treated for 1 or 2 weeks with 250 µg/ml of a monoclonal antibody against IL-15 (TEV-53408) as proof of concept. Treatment started either on day 21 or day 28 of the assay. IgG matching isotype control at the same concentration of 250 µg/ml was used as a negative control.

**Figure 4:**
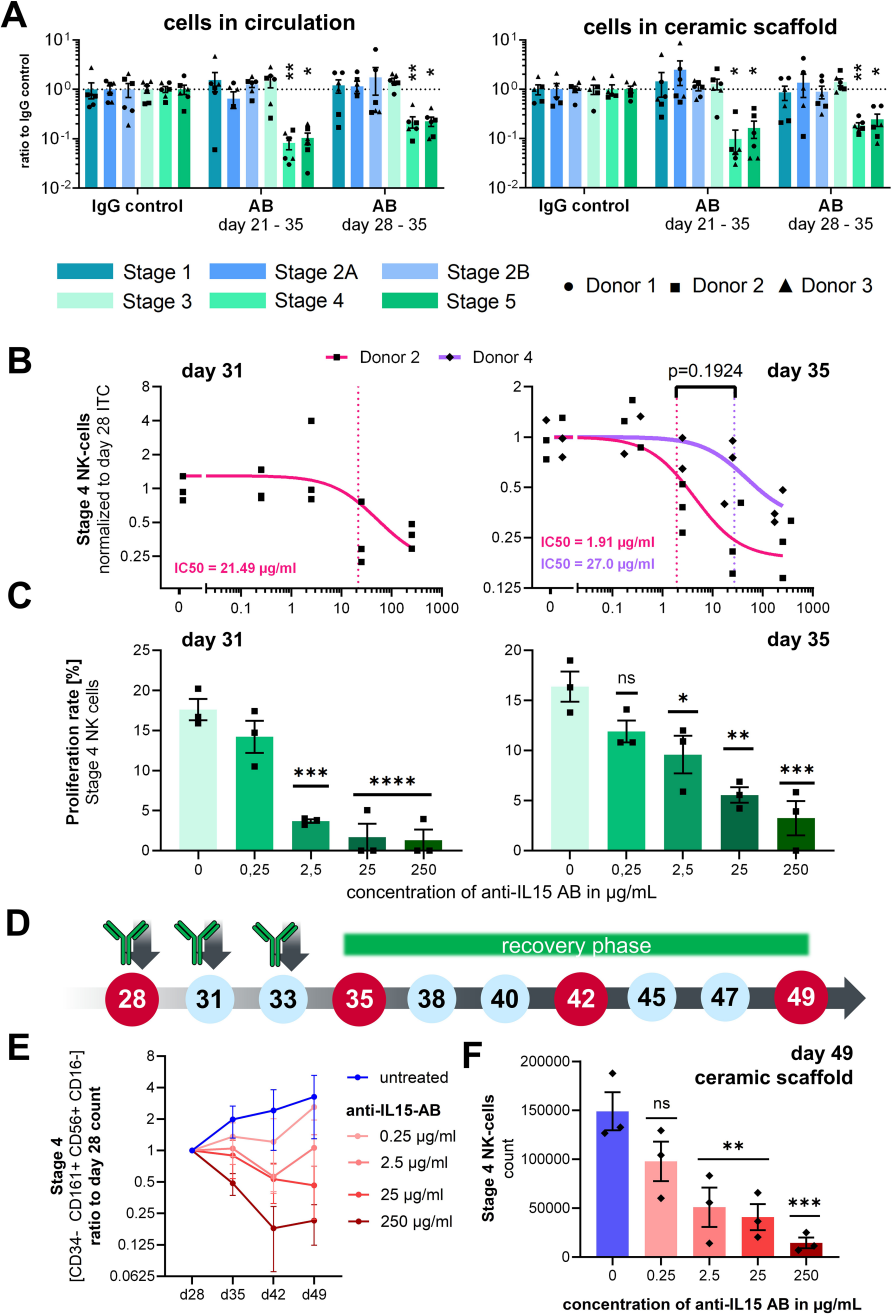
Effects of anti–IL-15 antibody TEV-53408 on stage specific NK cell counts. **(A)** Treatment effect on day 35 after 7 or 14 days of treatment with 250 µg/mL in the circulating cell pool and in the ceramic scaffold population. Mean values ± s.e.m. of three donors (N = 3) with two chips (n = 2) are shown. Raw cell counts were compared by a repeated measures mixed effects model REML with the Geisser-Greenhouse correction and Dunnett’s multiple comparisons test, n = 6 chips, **p < 0.01 *p < 0.05 and ns (p > 0.05). **(B)** Relative cell counts of stage 4 NK cells in circulation on days 31 and 35 over a concentration range of the anti–IL15-antibody from 0.25 to 250 µg/ml normalized to the stage 4 NK cells counts sampled from the same circuit on day 28. Individual circuits and mean values ± s.e.m. of two donors with three chips are shown (only one donor for day 31 analysis). Concentration response curves were calculated as a three parameters curve with a least squares regression. IC50 values were compared with an extra sum-of-squares F test. **(C)** Proliferation rate of stage 4 NK cells in circulation on day 31 and day 35 measured by EdU staining over a concentration range of the anti–IL-15 antibody from 0.25 ng/ml to 250 µg/ml. Mean values ± s.e.m of three chips (n = 3) from one donor are shown. Statistical comparison of means was performed by a one-way ANOVA plus Dunnett’s *post hoc* test with the IgG isotype condition as control condition, n = 3 chips, ****p < 0.0001, ***p < 0.001, **p < 0.01 *p < 0.05 and ns (p > 0.05). **(D)** Time schedule of the antibody application, cell sampling, and start of a recovery phase after day 35 of the assay. **(E)** Cell counts of Stage 4 NK cells in circulation sampled from untreated control circuits and circuits treated from day 28 to day 35 with different concentrations of the TEV-53408 antibody and allowed to recover following washout and medium exchange between day 35 to day 49. Measured cell counts were normalized to the cell counts in the same circuit at day 28. Mean values ± SD of three chips of one donor are shown. **(F)** Cell counts of Stage 4 NK cells in the ceramic scaffold sampled from untreated control circuits and circuits treated from day 28 to day 35 with different concentrations of the TEV-53408 antibody and allowed to recover following washout and medium exchange between day 35 to day 49. Mean values ± s.e.m. of three chips (n = 3) of one representative donor are shown. Statistical comparison of raw cell counts was performed by a one-way ANOVA plus Dunnett’s *post hoc* test with the IgG isotype condition as control condition, n = 3 chips, ***p < 0.001, **p < 0.01 *p < 0.05, and ns (p > 0.05).

On day 35, a significant reduction in stages 4 and 5 cell counts in the ceramic scaffold (**Figure 4B**) and the circulation (**Figure 4A**) in comparison to the IgG control was measured for all donors. Treatment effects were visible for chips treated for either one (days 28–35) or 2 weeks (days 21–35). All other NK cell progenitor populations were not affected by the application of the anti–IL-15 antibody.

CD34+ cell donor 2 and an additional donor 4 were used in two additional experiments to analyze the dose-dependency of the antibody effect in a range of 0.25 µg/ml, 2.5 µg/ml, and 25 µg/ml up to 250 µg/ml. Treatment with TEV-53408 was initiated on day 28, since the effect was similar between the 2 tested time points, and higher counts of stage 4 NK cells were detected on day 28. Cells were sampled from the medium compartment on days 28, 31, and 35 of the assay in the experiment with donor 2 and on days 28 and 35 in the experiment with donor 4.

Concentration-dependent treatment effects were visible on stage 4 NK cells at TEV-53408 concentrations of 2.5 µg/ml and higher after seven days of treatment (**Supplementary Figure 5D**). To take into account the underlying variability of NK cell counts on day 28 (**Supplementary Figure 5A**), stage 4 NK cell counts at days 31 and 35 (**Supplementary Figures 5C, D**) were normalized to the baseline counts of the same circuit on day 28 for calculation of the IC50. Dose-dependent reduction of stage 4 NK cell numbers in circulation was detected after seven days of treatment with the anti–IL-15 antibody for both tested donors. The difference between calculated IC50 values of both donors was not significant (**Figure 4C**). The dose-dependent treatment effect of the antibody increased over time as indicated by a lower IC50 value on day 35 (1.91 µg/ml) compared to day 31 (21.49 µg/ml) for donor 2 (**Figure 4C**).

EdU+ proliferating cells in the stage 4 NK cell population was measured in circulation samples on day 28, day 31, and day 35. The baseline frequency of proliferating stage 4 cells on day 28 was around 15% (**Supplementary Figure 5B**) and was significantly reduced (on day 31 after treatment with TEV-53408 concentrations of 2.5 µg/ml or higher to below 5% proliferation rate at the highest concentration. On day 35 higher proliferation frequencies were measured compared to day 31 but still significantly lower than in the control condition (**Figure 4C**). However, total numbers of stage 4 NK cells were reduced (**Supplementary Figure 5D**) showing that numbers of proliferating stage 4 NK cells on day 35 were still low in the treated circuits. Treatment-induced reduction of proliferation rate was not observed in the stage 2B progenitor population (**Supplementary Figure 4E**). This result is in line with the recognized role of IL-15 in NK cells maturation and proliferation in the bone marrow, and appears to provide a proof-of-concept that this bone marrow model is a clinically relevant framework for studying drug effects such as efficacy or toxicity in a physiologically relevant human microenvironment.

To determine whether the effect on NK cells is reversible, several chip circuits of HSC donor 4 were supplemented with fresh differentiation medium after the treatment with TEV-53408 was stopped on day 35, and the differentiation of NK cell stages was traced for two additional weeks (**Figure 4D**). Treatment with the TEV-53408 antibody had a clear concentration depended effect on Stage 4 NK cells throughout the treatment period and the recovery phase. Full reversibility was observed for lower doses on circulating cell population (**Figure 4E**) and in the ceramic scaffold (**Figure 4F**). For the two highest doses (25 and 250 µg/ml) full reversibility was not detected within the 2-week window long-lasting treatment effects were also detected in the analysis of stage 4 NK cells (**Figure 4F**) and stage 5 NK cells (**Supplementary Figure 7E**) from the ceramic scaffold, with significantly lower counts in circuits treated with doses of 2.5 µg/ml and higher.

NK cell counts were plotted relative to the counts of day 28 to account for the variability of different circuits. Counts of NK cell stages 1–3 in circulation fluctuated during the days 35–49 period. Lower counts of stages 2A and 2B NK cell progenitors were observed upon treatment with the anti–IL-15 antibody in the circulating cell population (**Supplementary Figure 6A**) and in the ceramic scaffold population on day 49 (**Supplementary Figures 7B, C**), although not with a clear concentration dependency. NK cell stages 4–6 counts increased further after day 28 in the untreated control group (**Figure 4E**; **Supplementary Figure 6E**).

Mean fluorescence intensity (MFI) of CD107a stage 4 NK cells was measured with and without PMA/ionomycin stimulation on day 35 of the assay after 7 days of treatment with the anti–IL-15 antibody and during the recovery period. Strong activation with almost a 100× increase of the MFI was observed on all time points from day 35 to day 49. No differences were detected between cells that were treated with the TEV-53408 antibody and untreated cells, indicating that the treatment did not affect the ability of the remaining stage 4 NK cells to be activated (**Supplementary Figure 8**).

## Discussion

Studying the development of human NK cells and understanding their differentiation in the bone marrow is essential for both basic research and clinical applications. There are several models, both *in vitro* and *in vivo*, that can be used to study NK cell differentiation in the bone marrow. While *in vivo* models such as genetically modified mice and humanized mice can provide a whole-organism context and allow for the study of systemic interactions and long-term effects, the differences between mouse and human immune systems may limit the direct applicability of findings to humans. In contrast, human-relevant *in-vitro* models, including 2D cultures, co-culture systems, and advanced 3D scaffolds or organ-on-a-chip technologies, offer precise control of experimental conditions and better mimic the bone marrow microenvironment, although they can be technically complex and costly. *Ex-vivo* models, such as bone marrow explants, preserve native architecture and cell interactions, providing a near-physiological context but are limited by their short lifespan and variability.

In this work, a microfluidic bone marrow model focused primarily on NK cell development has been established. Specifically, HSPCs from four different donors were seeded into a ceramic scaffold in the presence of human MSCs in a microfluidic device recapitulating the human bone marrow. NK cell differentiation was induced by the application of a lymphoid differentiation medium containing IL-15. NK cell development according to surface markers followed a common schema of human NK cell development (9). Expression of myeloid marker CD33 on early stage NK cell progenitors suggests that, in the HUMIMIC Chip2, NK cells are derived from granulo-myelomonocytic progenitors and do not develop exclusively from lymphoid progenitors. This differentiation pathway was characterized in a study of NK cell reconstitution in humanized mice showing that functional NK cells can develop from CD56^low^CD33^+^ myeloid NK precursors that lose CD33 expression during further differentiation (36). However, it is still in debate how well these results translate to the *in vivo* differentiation pathways of NK cells and whether myeloid-derived NK cells are an artifact of *in-vitro* culture (33).

Within 28 days of culture, all developmental stages of NK cell differentiation were detected in the HUMIMIC Chip2, although the counts of mature NK cells Stage 5 (CD56+CD16+) were low, indicating that the current conditions do not support efficient differentiation beyond stage 4. Since we did not further investigate the NK cell differentiation upon stimulation with additional growth factors, it cannot be excluded that certain factors that would allow further maturation are missing in the assay setup. Also, it was described before that NK cells derived from myeloid precursors *in vitro* are mostly of a CD56^bright^ phenotype with low CD16 expression (37). Progression into stage 5 could be partially achieved by the cultivation of cells past day 35. On day 49 of the assay, a distinct CD161^+^CD56^+^CD16^+^ fraction was observed in the ceramic scaffold. This phenotype resembles an NK cell phenotype known to develop *in vivo* after allogeneic hematopoietic stem cell transplantations. These cells were described to be present for up to one year post-transplantation and being fully functional, including degranulation activity upon stimulation (38). Cells identified as stage 4 NK cells in this study were found to be functional as shown by upregulation of CD107a upon stimulation with PMA and ionomycin, which is described to correlate with NK cell degranulation following stimulation (39). Overall, this model provides a human-relevant 3D system to study the kinetics of NK cell maturation in a perfused, *in vitro* bone marrow-like setting over multiple weeks while maintaining a pool of CD34+ stem and progenitor cells, which enables compound testing with complex repeated dosing and recovery schedules. In the future, the model can be further extended by modeling secondary lymphoid tissues to map the complete maturation pathway of NK cells. The model has the potential to be expanded into a general lymphoid assay system that also maps the development of ILCs, B cell, and T cell.

Interleukin-15 (IL-15) is a key cytokine for the progression of NK-cell differentiation from the progenitor stages into the CD56+ stages and subsequent maintenance of these cells (35). IL-15 binds to the IL-15 receptor, which consists of three subunits: the IL-15 receptor α, CD122, and CD132. Removal of IL-15 from the cell culture medium between day 28 and day 35 led to a reduction of stages 4–6 NK cells both in circulation and inside the ceramic scaffold compared to a control condition continuously supplemented with IL-15. Decreasing numbers of NK cells upon IL-15 withdrawal indicate the presence of the IL-15 receptor in these cells, contradicting missing CD122 expression indicated by FC staining.

The effect of TEV-53408 on NK cell counts was concentration-dependent and robust across different donors of CD34+ progenitor cells, independent of application timing and affecting the same CD56+ populations. Stage 4 NK cell numbers decreased by 62% (donor 4) to 77% (donor 2) in the highest concentration after 7 days of continuous treatment. The reduction of later stages of NK cell differentiation (stage 4, CD56+CD16-) by TEV-53408 was preceded by a reduction of the proliferation rate of these stages as shown by EdU+ staining. This indicates that TEV-53408 interferes with the maturation of NK cells due to inhibition of proliferation and not via apoptosis.

The reduction of stage 4 NK cells was also reversible in a dose-depe7ndent manner, 2 weeks after the treatment was stopped. For the recovery phase, it must be considered that complete removal of the antibody from the system is not possible in one step, since the cell culture medium inside the ceramic scaffold cannot be removed without disturbing the system. Therefore, slower recovery in the high-dose circuits could also be attributed to residual antibody in the system. Importantly, the treatment did not affect the functionality of the stage 4 NK cells, as CD107a expression could be induced upon stimulation at all time points independent of antibody treatment.

Observed effects were in line with data from a clinical trial with TEV-53408 (40) and those in macaques induced by TEV-53408 (37) and other anti–IL-15 antibodies (34).

*In vitro* cell-based assays to study the effect of investigational therapeutic antibodies on human NK cells usually employ NK cell lines (i.e., NK92, YTS, and NKL) or primary NK cells purified from peripheral blood. These cells are already expressing CD56 and hence mimic the NK cells of the peripheral blood and do not allow investigation of the effect of treatment on the NK development process taking place in the bone marrow. Similarly, the effect of therapeutic anti–IL-15 antibodies has been studied in animal models, yet the translatability of the results from animals to humans is limited by differences in the immune system between animals and humans and a lack of cross-reactivity of the antibodies (41). Suitable assays allowing *in vitro* assessment of human NK cell lineage development are missing, especially considering the ability to analyze effects on NK cell progenitors.

The presented proof-of-concept assay still has some limitations that should be addressed in future iterations of the setup. The FC panel used only allows for a fairly broad separation of NK subpopulations and does not take into account dendritic cells and other innate lymphoid cell populations that are likely to be co-developing in this setup. NK cell functionality was assessed only by analysis of post-stimulation CD107a marker expression but not also by cytotoxicity assays or cytokine quantification. While it is debatable whether functionality is expected for NK cells within the bone marrow niche, it would be interesting to investigate this question in future studies.

Taken together, this microfluidic human BM Chip recapitulates several key aspects of human NK cell differentiation and maturation and appears to be a complementary model to test the effect of drugs, such as anti–IL-15 inhibitors, at clinically relevant exposures. In the future, it might be possible to couple this system with other organ-on-chip models to study the effect on NK cells in secondary lymphoid organs and within the tumor microenvironment.

## Materials and methods

### Preculture of mesenchymal stromal cells

Cryopreserved human bone marrow stromal cells (Stemcell Technologies, Vancouver, BC, Canada) were thawed and cultured in MSC medium consisting of DMEM + 10% FCS + 1% penicillin-streptomycin (Corning, Corning, NY, USA). Stromal cells were cultured in T175 cell culture flasks at 37°C in a humidified atmosphere containing 5% CO2 with medium exchanges every 2–3 days until they reached 80%–90% confluence and were passaged using 0.05% Trypsin/0.44 mM EDTA (Corning, Corning, NY, USA). Cells were cultured for one or two passages before they were seeded onto the ceramic scaffold.

One day prior to the seeding of the cells, autoclaved ceramic scaffolds (TissUse GmbH, Berlin, Germany) were transferred to a 48-well plate and incubated in MSC medium overnight at 37°C. The next day, MSCs in monolayer culture were passaged, and an MSC seeding solution with a cell density of 2 × 10^6^ cells/ml was prepared. The cell culture medium was aspirated from the ceramic scaffolds before they were transferred into 96-well flat-bottom ULA plates using sterile forceps. To seed the MSCs onto the ceramic scaffold, 150 µl of the cell seeding solution was added to each scaffold/well and incubated for 5h. Afterwards, seeded scaffolds were cultivated for 10 days in 24-well ULA plates with medium exchanges every 2 to 3 days.

### Seeding CD34+ hematopoietic cells and chip culture

Cryopreserved human bone marrow CD34+ cells (Stemcell Technologies, Vancouver, BC, Canada) were thawed in IMDM + 10% FCS (Corning, Corning, NY, USA) according to the supplier´s manual and resuspended in chip culture medium consisting of Stemspan AOF (Stemcell Technologies, Vancouver, BC, Canada) supplemented with 10 ng/ml TPO, 25 ng/ml FLT-3L, 20 ng/ml SCF (all Peprotech, Cranbury, NJ, USA), 50 ng/ml IL-7, 50 ng/ml IL-15 (both Miltenyi Biotec, Bergisch Gladbach, Germany), 100 µg/ml holo-transferrin (Sigma-Aldrich, St. Louis, MO, USA), and 1% penicillin-streptomycin (Corning, Corning, NY, USA). The CD34+ cell seeding solution was adjusted to a density of 2.67 × 10^5^ living cells/ml. Ceramic scaffolds pre-seeded with stromal cells were transferred into 96-well ULA plates, and 150 µl of CD34+ cell seeding solution containing 40,000 CD34+ cells were applied to each ceramic. After 5h, ceramic scaffolds were transferred into the outer compartment of the HUMIMIC Chip2 (TissUse GmbH, Berlin, Germany) and cultured in a total volume of 800 µl chip culture medium. Microfluidic recirculation was initiated immediately by connecting the chips to the HUMIMIC Starter (TissUse GmbH, Berlin, Germany) with a pumping frequency of 0.5 Hz and pressure/vacuum settings of ±300 mbar.

### Dynamic cultivation in the bone marrow chip

Medium was exchanged every second to third day, with or without cell sampling. Cells were sampled for FC analysis from the medium compartment once per week. On days without cell sampling, 200 µl were withdrawn from each compartment and replaced with fresh chip culture medium. Sampled medium was centrifuged for 5 min at 300 g, and the resulting supernatant was used for LDH and glucose analysis.

To extract cells for FC analysis, cell culture medium in the medium compartment was thoroughly resuspended, and the cell suspension was transferred into 1.5 ml centrifugation tubes that were stored on ice until FC staining. The sampled medium was replaced with 400 µl of fresh chip culture medium.

### Preparation of chip cultivation medium and treatment media

Chip cultivation medium was prepared fresh on every medium exchange day. Growth factors were stored in aliquots at −20°C and added to the chip cultivation medium on every medium exchange day. On the specified day when the treatment with the anti–IL-15 antibody TEV-53408 (Teva Pharmaceutical Industries, Petach Tikwa, Israel) was started, the antibody was added during the medium preparation. At the first treatment day, a 100% medium exchange was performed sampling the complete medium from the ceramic and the medium compartment. All following exchanges were performed, as a 50% medium exchange as described before.

### Cell sampling from the ceramic scaffold

On the final day of the study, hematopoietic cells were also sampled from the ceramic scaffold model. First, suspension cells were sampled from the medium compartment as described before. Then, the supernatant from the ceramic compartment was sampled and carefully transferred into a 5-ml centrifugation tube, further referenced as the ceramic sample tube. Following this, all inserts were removed from the chip, and the ceramic scaffold was transferred into a 24-well plate containing 1.2-ml of Phosphate Buffered EDTA (PBE) buffer, consisting of phosphate-buffered saline (PBS) plus 0.6% bovine serum albumin (Sigma-Aldrich, St. Louis, MO, USA) and 1 mM EDTA (Corning, Corning, NY, USA). The ceramic scaffold was then thoroughly flushed with PBE, with the subsequent transfer of the extracted cell suspension into the ceramic sample tube. The ceramic scaffold was then relocated to a new well within the 24-well plate, where it underwent a 10-min incubation at room temperature in 1.2 ml of PBE. In the meantime, each insert was washed twice with 300 µl of PBE, and the resulting washing solution was transferred into the ceramic sample tube. To ensure comprehensive cleaning, the microfluidic channels of Chip2 were flushed with 1 ml of PBE using syringes. After the 10-min incubation period, the ceramic scaffold underwent a second flush with PBE, and the resulting washing solution was transferred into the ceramic sample tube.

### Labeling of proliferating cells by 5-ethynyl 2´-deoxyuridine

In experiments incorporating labeling of proliferating cells with 5-ethynyl 2´-deoxyuridine (EdU), the harvested cell suspension was not stored in 1.5 ml centrifugation tubes but instead directly transferred into 24-well ULA plates, which were stored on a 37°C heating plate inside of the clean bench during the sampling procedure. After all chips were finished, 10 µM EdU (Thermo Fisher Scientific, Waltham, MA, USA) was added, and the cell suspensions were incubated for 1h at 37°C. Afterward, samples were transferred into 1.5 mL tubes and processed as described before.

### Cell count after cell sampling

Sampled cells from the medium circulation or the ceramic scaffold were centrifuged for 5 min at 300*g*, and the cell pellet was resuspended in 500 µl of FC buffer consisting of PBS + 3% FCS (Corning, Corning, NY, USA). For measurement of cell count and viability, cells were counted with an automated cell counter, NucleoCounter NC-200 (ChemoMetec GmbH, Kaiserslautern, Germany), measuring total cell count, living cell count, and viability.

### NK-cell stimulation assay with phorbol 12-myristat 13-acetat and ionomycin

For the experiment involving NK-cell stimulation, a fraction of the cell sample was transferred into a 96-well U-bottom plate and was centrifuged at 300*g* for 5 min. The supernatant was discarded, and the cell pellets were resuspended in 300 µl StemSpan AOF supplemented with 0.2% eBioscience Cell Stimulation Cocktail (Thermo Fisher Scientific, Waltham, MA, USA). The plates were then left for incubation at 37°C for 2h. Afterwards, plates were again centrifuged at 300 g for 5 min, the supernatant was discarded, the cell pellet resuspended in FC buffer, and used for FC staining.

### Flow cytometry staining

After cell counting, cells were transferred into a 96-well round-bottom plate, and dead cells were stained with the LIVE/DEAD Fixable Yellow Dead Cell Stain Kit (Thermo Fisher Scientific, Waltham, MA, USA) for 20 min at 4°C. Afterwards, cells were blocked for 20 min at 4°C with Human TruStain FcX blocking solution (Biolegend, San Diego, CA, USA). The antibody staining cocktail was freshly prepared every time and consisted of CD161-BV421 (1:40, Clone HP-3G10, BioLegend, San Diego, CA, USA 339914), CD45-BV510 (1:40, Clone HI30, BioLegend, San Diego, CA, USA 304036), CD33-BV605 (1:20, Clone P67.6, BioLegend, San Diego, CA, USA 366612), CD16-FITC (1:40, Clone 3G8, BioLegend, San Diego, CA, USA 302006), CD122-FITC (1:40, Clone TU27, BioLegend, San Diego, CA, USA 339006), CD10-PerCp-Cy5.5 (1:20, Clone HI10a, BioLegend, San Diego, CA, USA 312216), CD56-PE-Cy7 (1:80, Clone 5.1H11, BioLegend, San Diego, CA, USA 362510), CD127-APC (1:20, Clone A019D5, BioLegend, San Diego, CA, USA 351316) and CD34-APC-Cy7 (1:80, Clone 561, BioLegend, San Diego, CA, USA 343614).

For experiments incorporating staining of EdU or staining of CD107a after PMA/ionomycin stimulation, an altered staining panel was used. Here, CD161-BV421, CD45-BV510, and CD122-FITC were removed from the panel and replaced by CD161-PE (1:40, Clone HP-3G10, BioLegend, San Diego, CA, USA 339904) and CD107a-BV421 (1:40, Clone H4A3, BioLegend, San Diego, CA, USA 328626).

The antibody staining cocktail for analysis of dendritic and myeloid cell populations consisted of CD1c-BV421 (1:40, Clone L161, BioLegend, San Diego, CA, USA 331526), CD33-BV605 (1:20, Clone P67.6, BioLegend, San Diego, CA, USA 366612), CD45-FITC (1:40, Clone 2D1, BioLegend, San Diego, CA, USA 368508), CD66b-PE (1:40, Clone G10F5, BioLegend, San Diego, CA, USA 305106), CD56-PE-Cy7 (1:80, Clone 5.1H11, BioLegend, San Diego, CA, USA 362510), CD141-APC (1:40, Clone M80, BioLegend, San Diego, CA, USA 344124 and CD14-APC-Cy7 (1:40, Clone M5E2, BioLegend, San Diego, CA, USA 301820).

Cells were incubated with the antibody cocktail for 20 min at 4°C and subsequently washed with FC buffer. For EdU staining, cells were fixed with 4% formaldehyde cells for 15 min at room temperature and resuspended afterwards in PBS+ 1% BSA. Staining of incorporated EdU was performed with the Click-iT Plus EdU Pacific Blue Flow Cytometry-Assay-Kit (Thermo Fisher Scientific, Waltham, MA, USA) according to the manufacturer’s instructions.

For all days, single staining controls and fluorescence minus one (FMO) controls were prepared. Stained samples were measured at a MACSQuant Analyzer 16 flow cytometer (Miltenyi Biotec, Bergisch Gladbach, Germany). Compensation was performed in FlowJo (BD) based on single staining controls and single staining bead controls. Gating was performed based on fluorescence minus one controls.

Dead events, debris and cell doublets were removed from the analysis. Then cells were separated into CD34^pos^/CD16^neg^ progenitor and non-progenitor population (CD34^neg^ and CD34^pos^/CD16^pos^ cells). CD34^pos^/CD16^neg^ cells were separated based on their CD10 and CD127 expression into non-lymphoid HSPC (CD10^neg^/CD127^neg^), stage 1 NK cells (CD10^pos^/CD127^neg^), stage 2A NK cells (CD10^pos^/CD127^pos^) and stage 2B NK cells (CD10^neg^/CD127^pos^). The non-progenitor population was separated into CD161^pos^ and CD161^neg^ events. CD161^pos^ events were separated based on their CD56 and CD16 expression into stage 3 NK cells (CD56^neg^), stage 4 NK cells (CD56^pos^/CD16^neg^), and stage 5 NK cells (CD56^pos^/CD16^pos^). CD161 negative cells were separated based on their CD33 and CD56 expression into unidentified NK-like cells (CD33^neg^/CD56^pos^), myeloid cells (CD33^pos^), and unspecified differentiated cells (CD33^neg^/CD56^neg^).

The relative fraction of each population of the overall living cell population was exported as readout. This percentage was multiplied with the previously acquired living cell count of the respective sample to calculate the total number of cells of every cell population in that sample.

### Statistical analysis

Results are presented as mean ± s.e.m. or ± SD as indicated in the figure legends. Statistical analyses were performed using GraphPad Prism 10 (GraphPad Software Inc., San Diego, CA, USA). Different levels of significance (*p*-values) are indicated by asterisks in each figure (*p < 0.05, **p < 0.01, ***p < 0.001, ****p < 0.0001).

Figure legends indicate the number of biological samples (*N*) of individual CD34+ cell donors and the number of individual independent chips (*n*).

Statistical comparison of means was performed using multiple t-tests (**Figure 3A**), repeated measures one- or two-way ANOVA (**Figures 3F**; **4C, F**), or a repeated measures mixed effects model REML (**Figures 3D**; **4A**). Following ANOVA, Dunnett’s multiple comparison test was used to compare each treatment group with the control group, and Tukey’s test was used for pairwise comparisons among all treatment groups to control for type I error.

## Data Availability

The raw data supporting the conclusions of this article will be made available by the authors, without undue reservation.

## Supplementary material

Supplementary Figure 1 Last iteration of the used flow cytometry gating tree, to separate Stages 1, 2, 3, 4, and 5 NK cells.

Supplementary Figure 2 Sampled cell counts of circuits over time from the circulation at the end of the first experiment. Mean values ± s.e.m of sampled living cells from three CD34+ donors with two chips (n = 2).

Supplementary Figure 3 Expression of CD34, CD10, CD127, CD161, CD56, CD16, CD122, CD33 and CD45 in events identified as stages 1, 2A, 2B, 3, 4, and 5 of NK cell development in primary bone marrow mononuclear cells **(A)** and sampled form the HUMIMIC Chip2 bone marrow model on day 28 of the assay **(B)**.

Supplementary Figure 4
**(A)** Mean fluorescence intensity of CD107a-BV421 on stages 1 and 2A NK cells on day 35 with and without PMA/ionomycin stimulation. Chip-specific intensity values and mean ± s.e.m. are shown from one experiment with two CD34+ donor with three to six chips (N = 2, n = 3-6). **(B)** Gating according to CD10 expression strength of stages 1 and 2A events into CD10low and CD10 high events and analysis of CD107a expression upon PMA/ionomycin activation in these subpopulations.

Supplementary Figure 5 Cell count of stage 4 NK cells in circulation on day 28 **(A)**, day 31 **(C)**, and day 35 **(D)** over a concentration range of the anti–IL-15 antibody. Proliferation rate of stage 4 NK cells in circulation on day 28 before treatment start (B) and of stage 2B NK cells in circulation on days 28, 31, and 35 over a concentration range of the anti–IL-15 antibody. Mean values ± s.e.m. of three chips (n = 3) are shown.

Supplementary Figure 6 Cell counts of stages 1 **(A)**, 2A **(B)**, 2B **(C)**, 3 **(D)** and 5 **(E)** NK cells in circulation sampled from untreated control circuits and circuits treated from day 28 to day 35 with different concentrations of the TEV-53408 antibody and allowed to recover without antibody application from day 35 to day 49. Measured cell counts of the different populations were normalized to the cell counts in the same circuit on day 28. Mean values SD of three chips (n = 3) are shown.

Supplementary Figure 7 Cell counts of stages 1 **(A)**, 2A **(B)**, 2B **(C)**, 3 **(D)**, and 5 **(E)** NK cells in the ceramic scaffold sampled from untreated control circuits and circuits treated from day 28 to day 35 with different concentrations of the TEV-53408 antibody and allowed to recover without antibody application from day 35 to day 49. Mean values ± s.e.m. of three chips (n = 3) are shown.

Supplementary Figure 8 CD107a mean fluorescence intensity of stage 4 NK cells in circulation on days 35, 42, and 49 and in the ceramic scaffold versus concentration of the anti–IL-15 antibody. Mean values ± s.e.m. of three chips (n = 3) before and after stimulation with PMA/ionomycin are shown.
