# Supplementary material for: Modeling human natural killer cell development and drug response in a microfluidic bone marrow model
Source: Front Immunol. 2025 Feb 20;16:1499397. doi: 10.3389/fimmu.2025.1499397 (PMC11883826; doi:10.3389/fimmu.2025.1499397)
Supplement: Supplementary Figure 1 — Last iteration of the used flow cytometry gating tree, to separate Stages 1, 2, 3, 4, and 5 NK cells. [file DataSheet1.docx]

Supplementary data


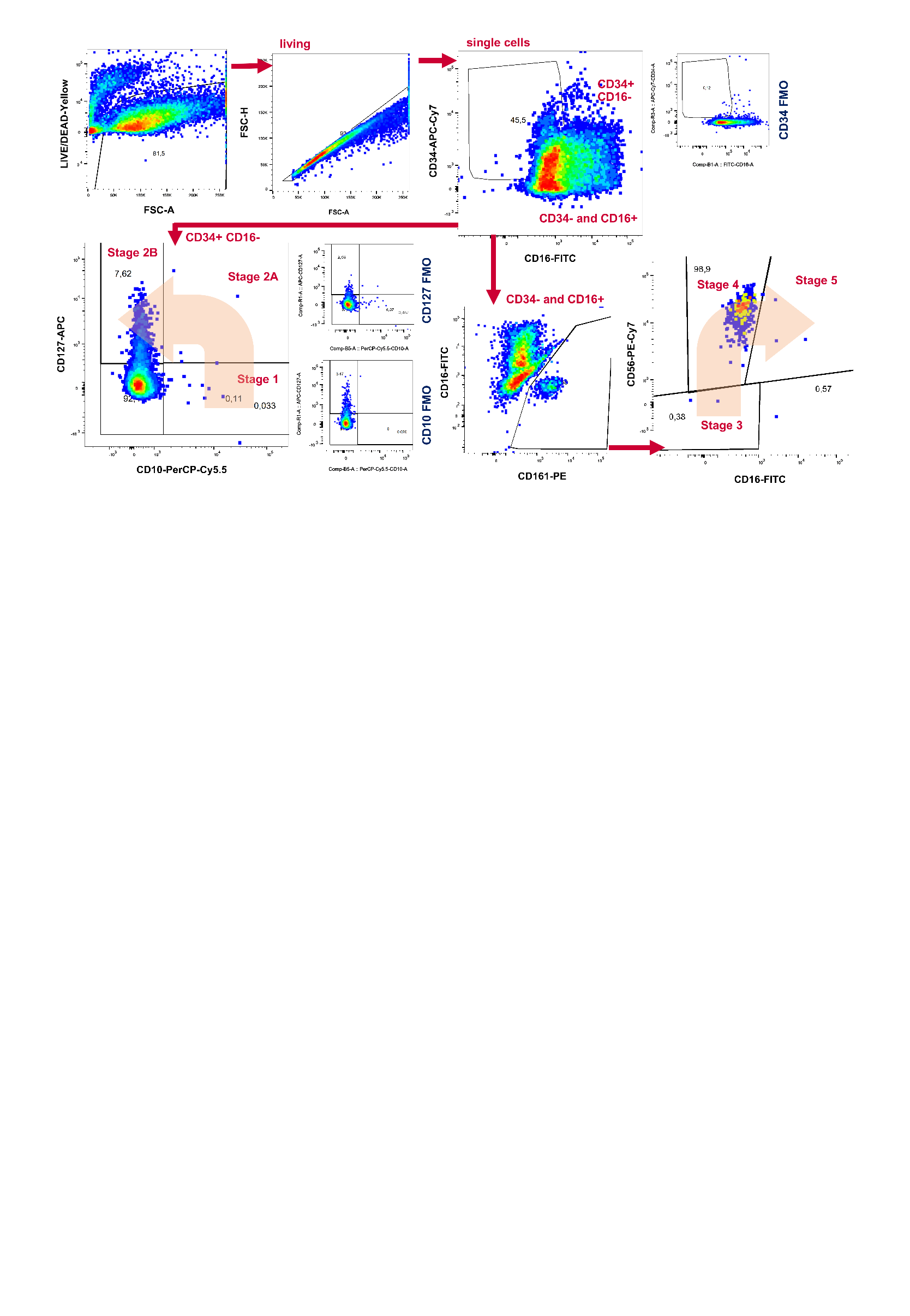


Supplementary Figure S 1: Last iteration of the used flow cytometry gating tree, to separate Stage 1, 2, 3, 4 and 5 NK-cells.


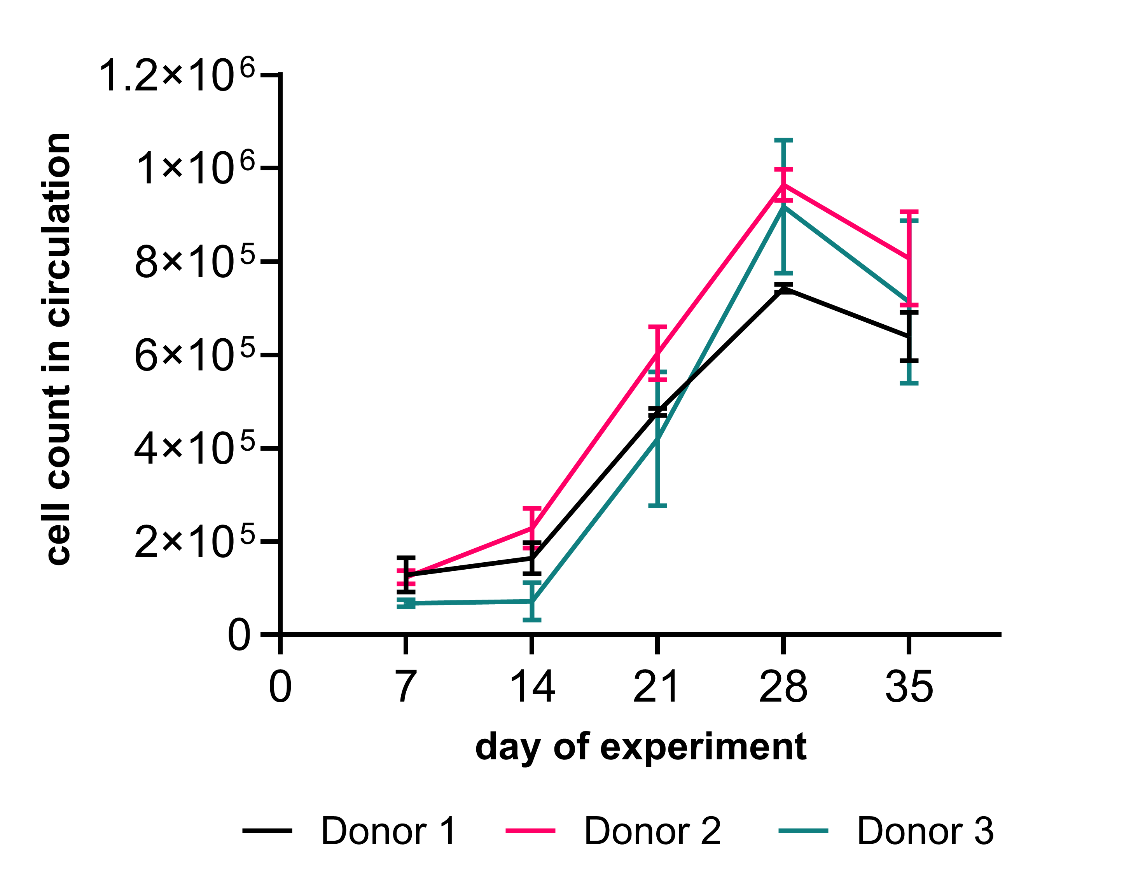


Supplementary Figure S 2: Sampled cell counts of circuits over time from the circulation at the end of the first experiment. Mean values ± s.e.m of sampled living cells from three CD34+ donors with two chips (n=2).


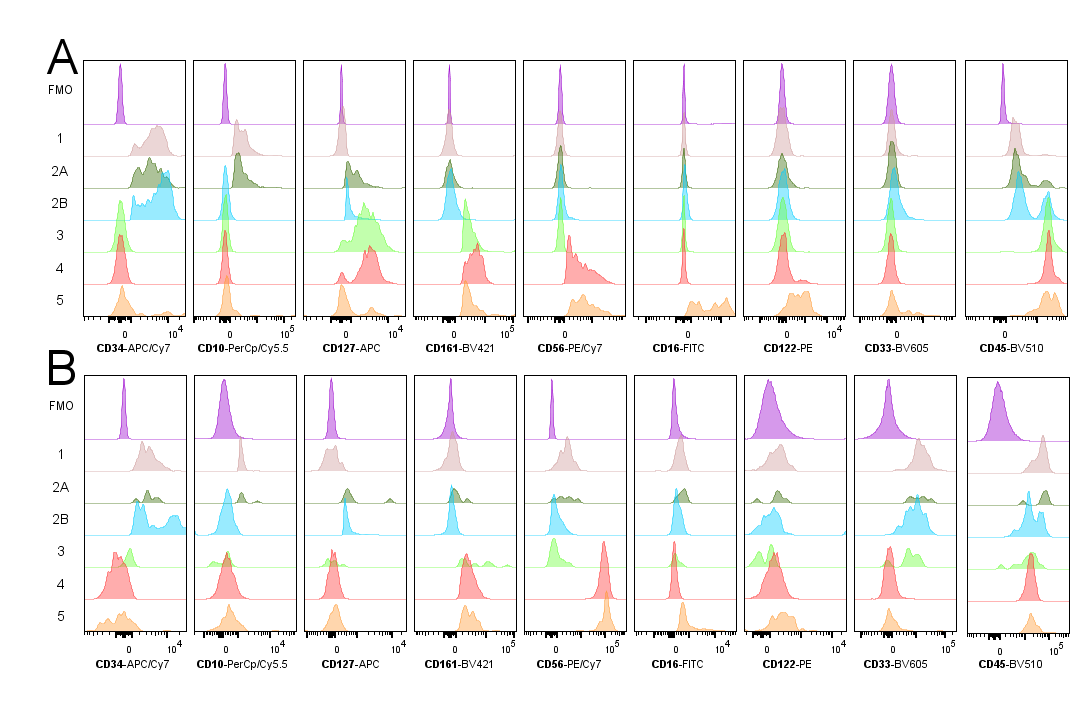


Supplementary Figure S 3: Expression of CD34, CD10, CD127, CD161, CD56, CD16, CD122, CD33 and CD45 in events identified as Stage 1, 2A, 2B, 3, 4 and 5 of NK-cell development in primary bone marrow mononuclear cells (A) and sampled form the HUMIMIC Chip2 bone marrow model on day 28 of the assay (B).


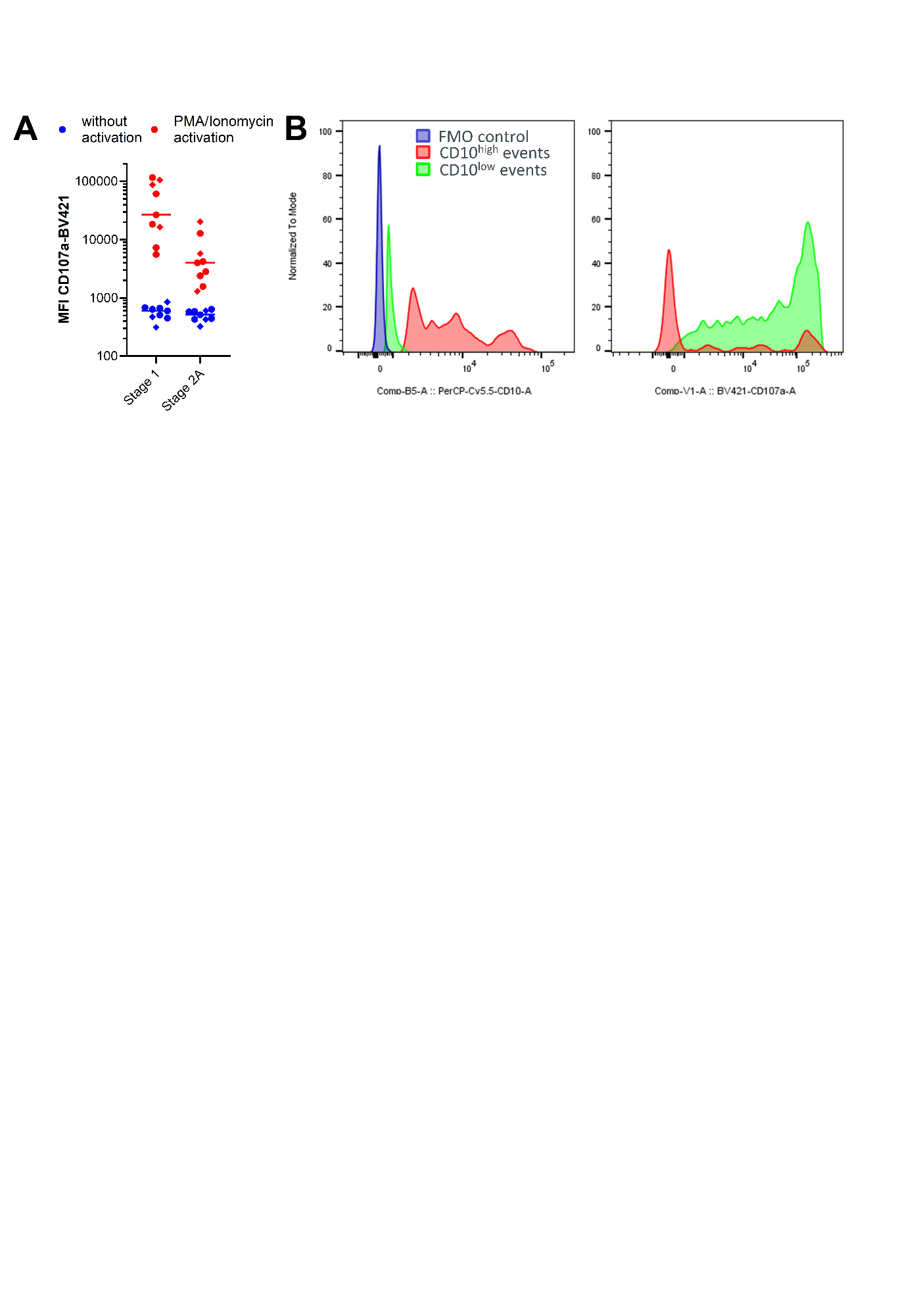


Supplementary Figure S 4: A: Mean fluorescence intensity of CD107a-BV421 on Stage 1 and Stage 2A NK-cells on day 35 with and without PMA/ionomycin stimulation. Chip-specific intensity values and mean ± s.e.m. are shown from one experiment with two CD34+ donor with three to six chips (N=2, n=3-6). B: Gating according to CD10 expression strength of Stage 1 and Stage 2A events into CD10low and CD10 high events and analysis of CD107a expression upon PMA/ionomycin activation in these subpopulations.


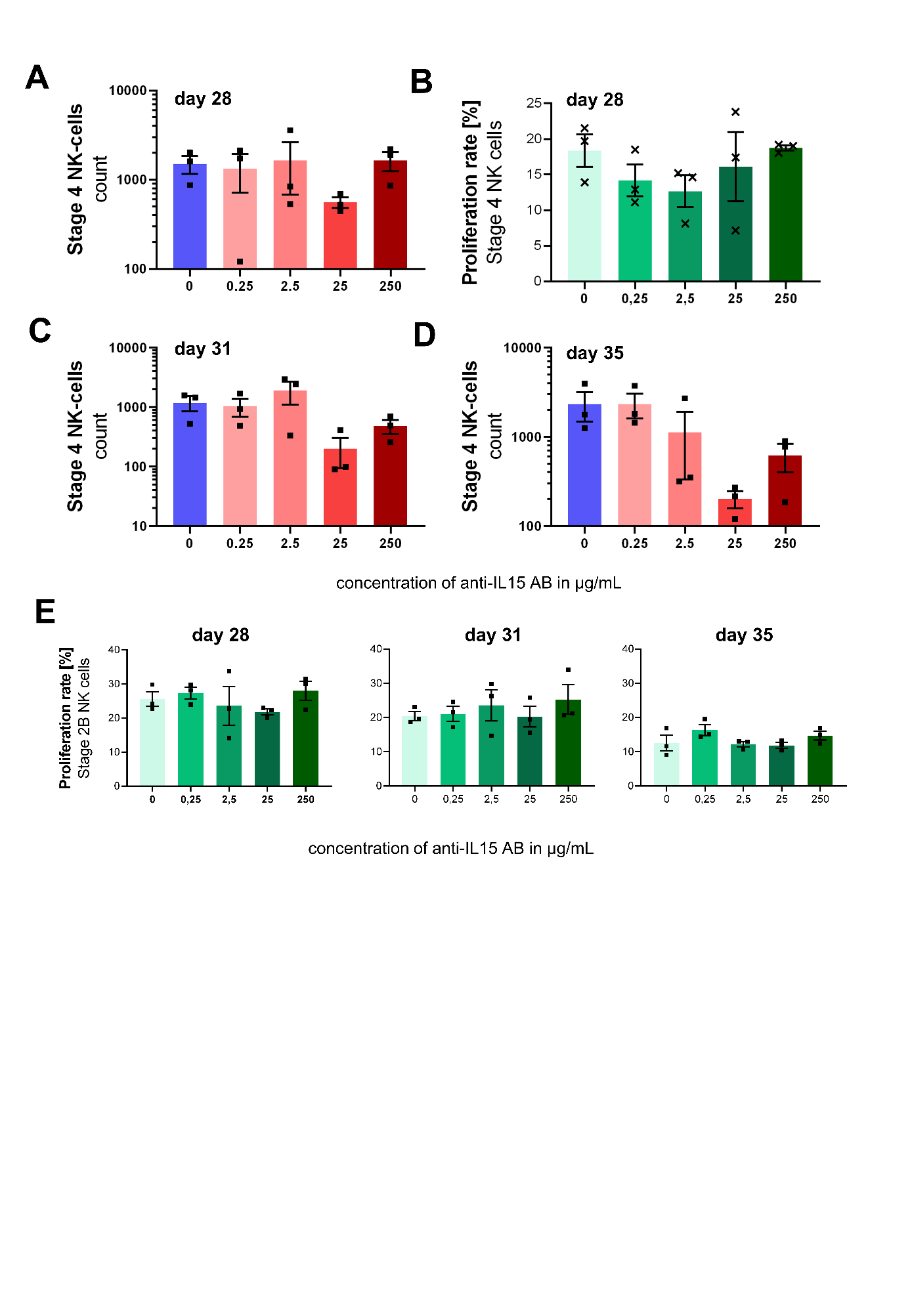


Supplementary Figure S 5: Cell count of stage 4 NK-cells in circulation on day 28 (A), day 31 (C) and day 35 (D) over a concentration range of the anti-IL15 antibody. Proliferation rate of stage 4 NK-cells in circulation on day 28 before treatment start (B) and of stage 2B NK-cells in circulation on day 28, day 31 and day 35 over a concentration range of the anti-IL15 antibody. Mean values ± s.e.m. of three chips (n=3) are shown.


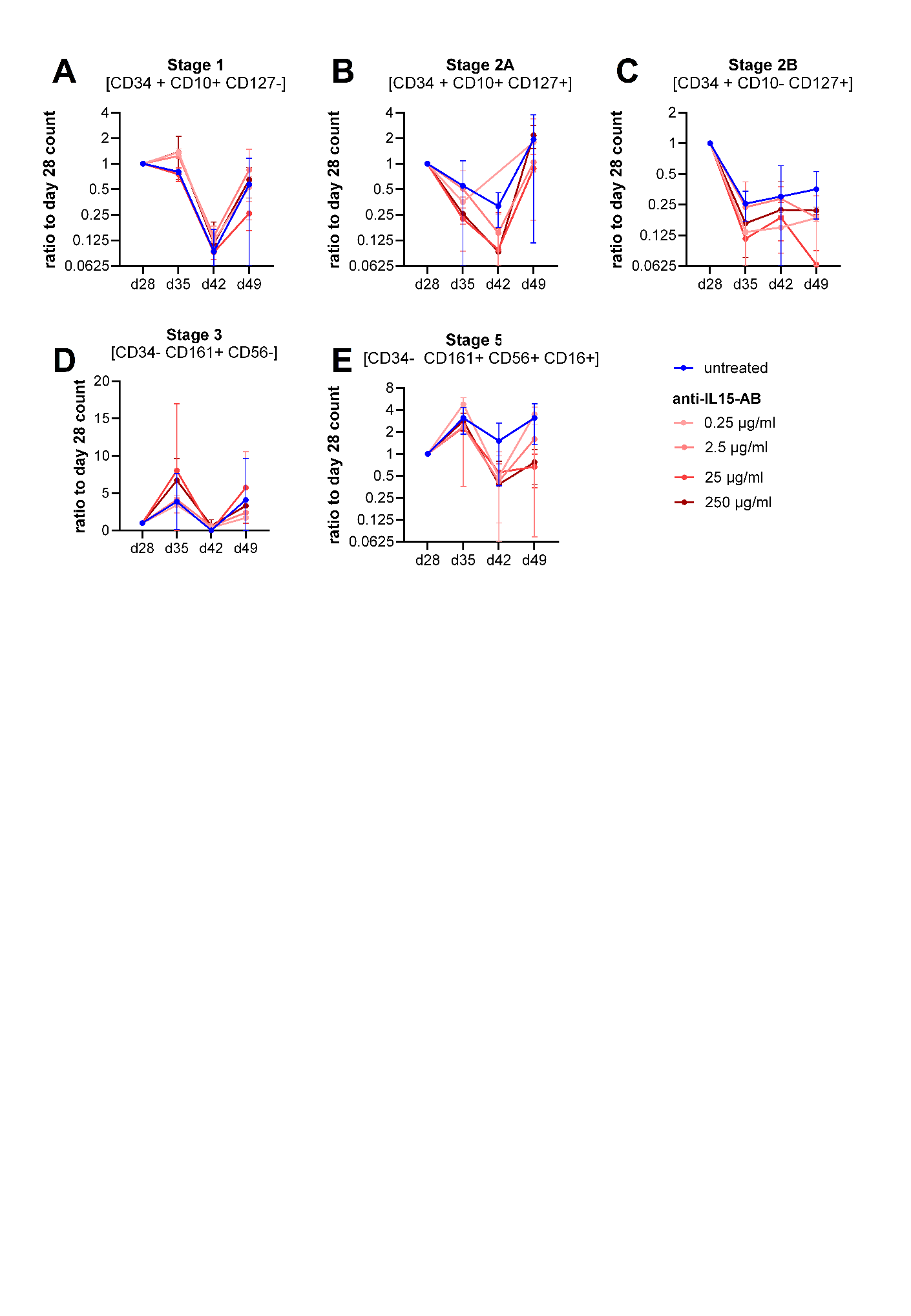


Supplementary Figure S 6: Cell counts of Stage 1 (A), Stage 2A (B), Stage 2B (C), Stage 3 (D) and Stage 5 (E) NK-cells in circulation sampled from untreated control circuits and circuits treated from day 28 to day 35 with different concentrations of the TEV-53408 antibody and allowed to recover without antibody application from day 35 to day 49. Measured cell counts of the different populations were normalized to the cell counts in the same circuit on day 28. Mean values SD of three chips (n=3) are shown.


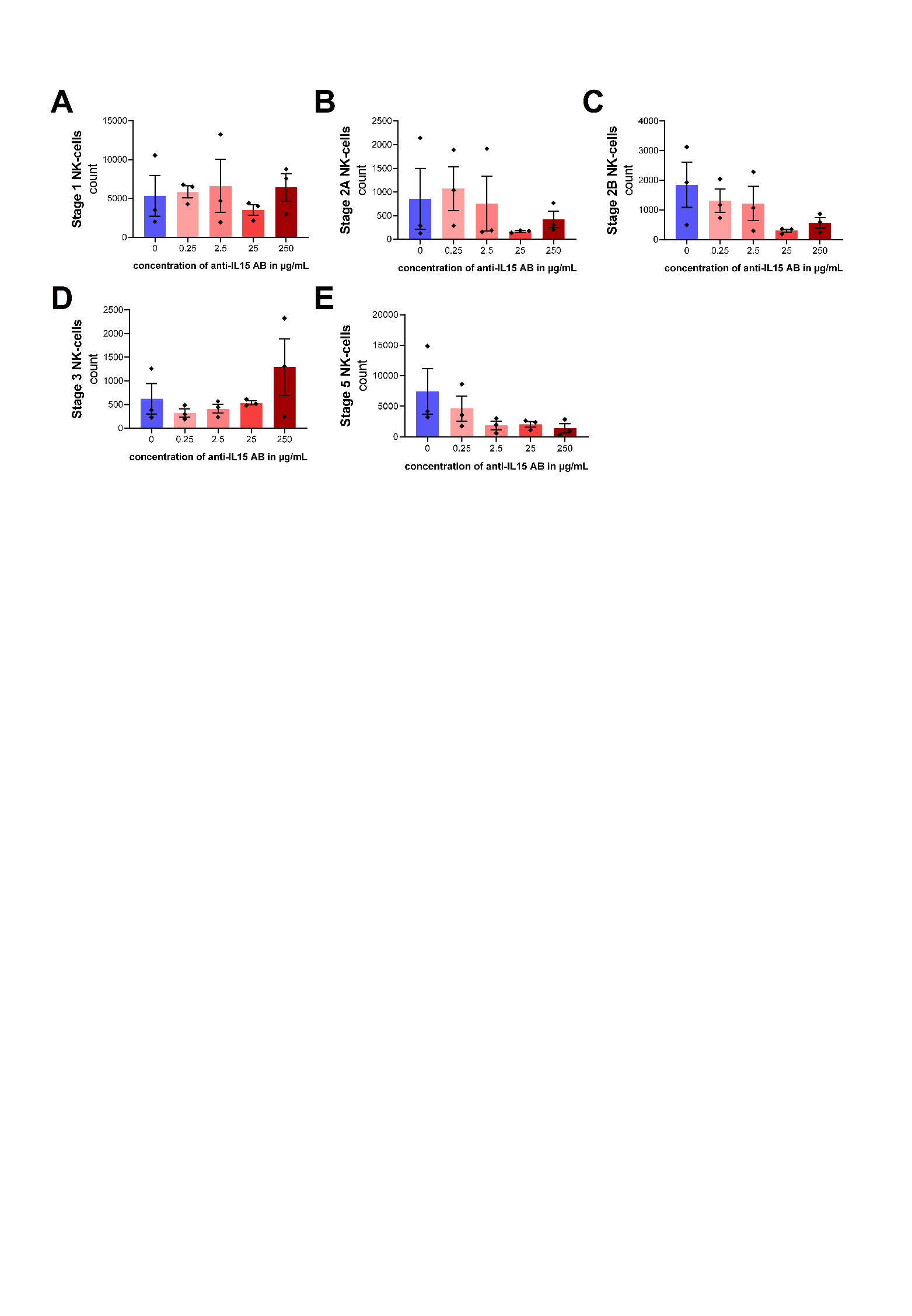


Supplementary Figure S 7: Cell counts of Stage 1 (A), Stage 2A (B), Stage 2B (C), Stage 3 (D) and Stage 5 (E) NK-cells in the ceramic scaffold sampled from untreated control circuits and circuits treated from day 28 to day 35 with different concentrations of the TEV-53408 antibody and allowed to recover without antibody application from day 35 to day 49. Mean values ± s.e.m. of three chips (n=3) are shown.


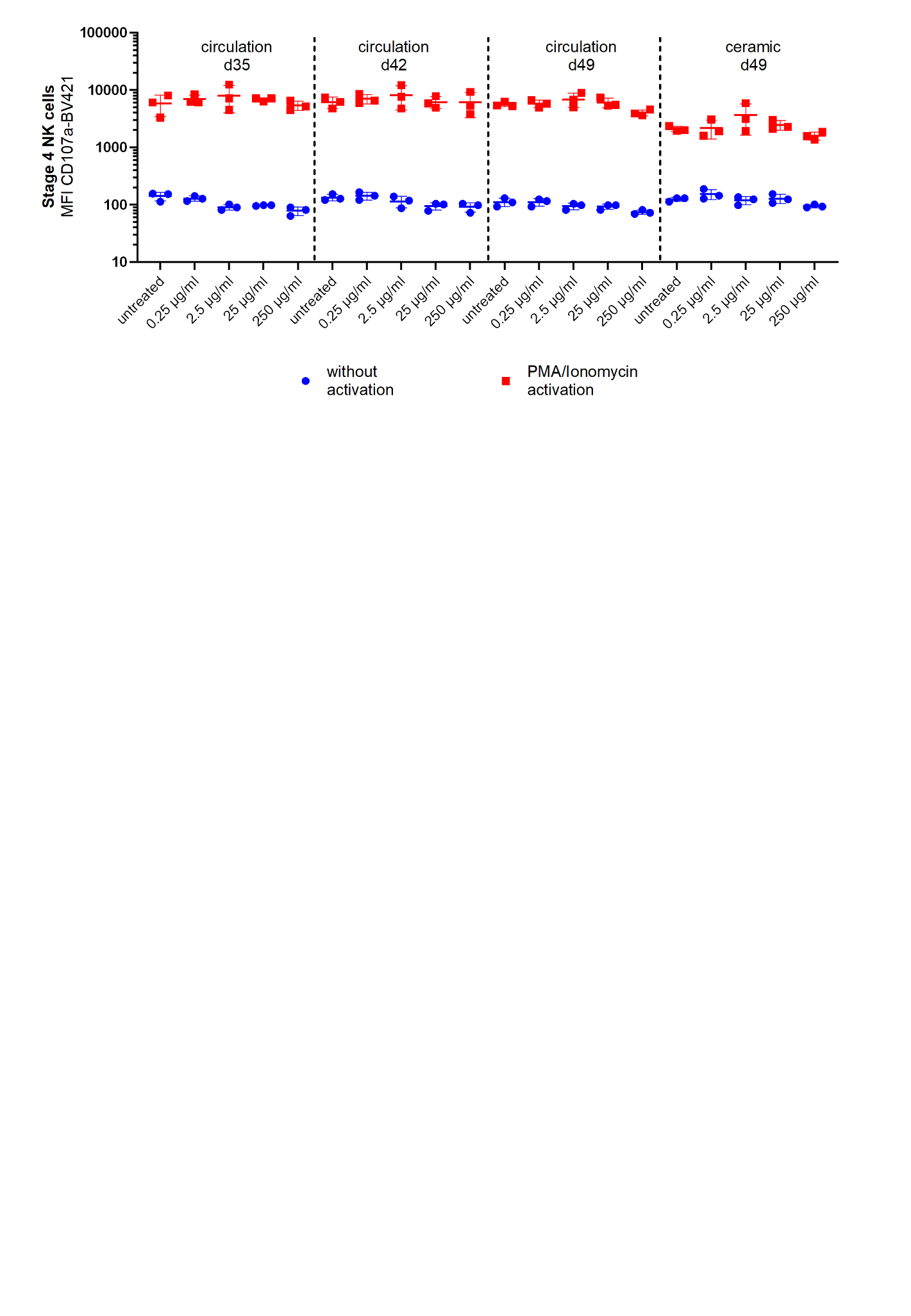


Supplementary Figure S 8: CD107a mean fluorescence intensity of Stage 4 NK cells in circulation on day 35, day 42, day 49 and in the ceramic scaffold versus concentration of the anti-IL15 antibody. Mean values ± s.e.m. of three chips (n=3) before and after stimulation with PMA / ionomycin are shown.
